# Systemic sclerosis is a risk factor of incident psoriasis: results from a nationwide cohort study

**DOI:** 10.3389/fimmu.2023.1326298

**Published:** 2023-12-14

**Authors:** Oh Chan Kwon, Kyungdo Han, Min-Chan Park

**Affiliations:** ^1^ Division of Rheumatology, Department of Internal Medicine, Yonsei University College of Medicine, Seoul, Republic of Korea; ^2^ Department of Statistics and Actuarial Science, Soongsil University, Seoul, Republic of Korea

**Keywords:** psoriasis, risk factor, systemic sclerosis, epidemiology, cohort

## Abstract

**Objective:**

Although the co-existence of systemic sclerosis (SSc) and psoriasis (PsO) has been reported, the risk relationship between the two diseases remains unclear. We aimed to assess whether SSc is associated with the risk of incident PsO.

**Methods:**

From the Korean National Health Insurance Service database, 4,933 patients with SSc and 24,665 age- and sex-matched controls were selected. Hazard ratios (HRs) and 95% confidence intervals (CIs) for incident PsO were estimated using multivariable Cox proportional hazard models adjusted for known risk factors of PsO. Further, we selected individuals whose health check-up data were available (2,355 patients with SSc and 11,775 age- and sex-matched controls). In this population, we further adjusted for additional risk factors of PsO using the health check-up data.

**Results:**

In the analysis of 4,933 patients with SSc and 24,665 age- and sex-matched controls, incidence rates of PsO in patients with SSc and controls were 10.26 and 3.20 per 1,000 person-years, respectively. After adjusting for risk factors of PsO, patients with SSc had a significantly higher risk of incident PsO (adjusted HR: 3.055 [95% CI: 2.597, 3.594]). Moreover, in the analysis of individuals who had health check-up data, additional risk factors of PsO were further adjusted; the result also showed that patients with SSc have a significantly higher risk of incident PsO (adjusted HR: 2.820 [95% CI: 2.207, 3.603]).

**Conclusion:**

Patients with SSc had a 3-fold higher risk of developing incident PsO than controls, independent of known risk factors of PsO.

## Introduction

Systemic sclerosis (SSc) is a rare autoimmune disease characterised by vasculopathy and fibrosis of the skin and internal organs (1, 2). Although the organs affected highly vary among patients with SSc, the skin is almost always affected (1). The skin involvement causes substantial morbidities, such as depigmentation, open ulcers, and pruritus, markedly diminishing the quality of life (3, 4). Moreover, extensive or rapidly progressing skin involvement is associated with progressive internal organ involvement, resulting in increased mortality (5).

Psoriasis (PsO) is an immune-mediated inflammatory disease that affects the skin (6, 7). Interestingly, anecdotal cases of co-existing SSc and PsO have been reported, suggesting a possible association between the two conditions (8). The possible link between SSc and PsO has been further evaluated in a cohort study from Israel (9). In that study, 2,431 patients with SSc were compared with 12,710 age- and sex-matched controls. PsO was more common in patients with SSc (1.9%) than in controls without SSc (1.2%). After adjusting for age, sex, body mass index (BMI), socioeconomic status, and smoking, patients with SSc had a 2-fold higher risk of having PsO (odds ratio, 2.16) than those without SSc (9). Although the study suggested SSc as a risk factor of PsO, PsO preceded SSc in 52.4% of the cases of co-existing SSc and PsO. This makes it difficult to conclude that SSc is a risk factor of incident PsO. Therefore, although the possible association between SSc and PsO is convincing by the large study population included in that study, it remains unclear whether SSc contributes as a risk factor for incident PsO. Moreover, other known risk factors of PsO, such as hypertension, diabetes, and dyslipidaemia (10), were not adjusted, which makes the risk relationship between the two diseases even more unclear.

To assess whether SSc contributes as a risk factor of incident PsO, we conducted a nationwide cohort study and estimated the risk of incident PsO in patients with SSc, compared with age- and sex-matched controls without SSc, and adjusted for multiple risk factors of PsO.

## Methods

### Data source

Data were extracted from the Korean National Health Insurance Service (NHIS) claims database. Comprehensive data such as demographics, socioeconomic status, medical treatments and procedures, disease diagnoses according to the International Classification of Diseases-10^th^ Revision (ICD-10) code, and rare intractable disease (RID) code (11) are included in the NHIS database. In the Korean RID registration system, a code for a specific rare disease is given based on a uniform diagnostic criteria provided by the NHI. Before registration, the fulfilment of the diagnostic criteria is thoroughly reviewed by the NHI and the corresponding healthcare institution. The profile of the data source has been described in detail previously (12). This study was approved by the Institutional Review Board (IRB) of Gangnam Severance Hospital (IRB No: 3-2022-0338). Owing to the retrospective nature of this study, the requirement for informed consent was waived and approved by the IRB of Gangnam Severance Hospital.

### Study cohort

**Figure 1:**
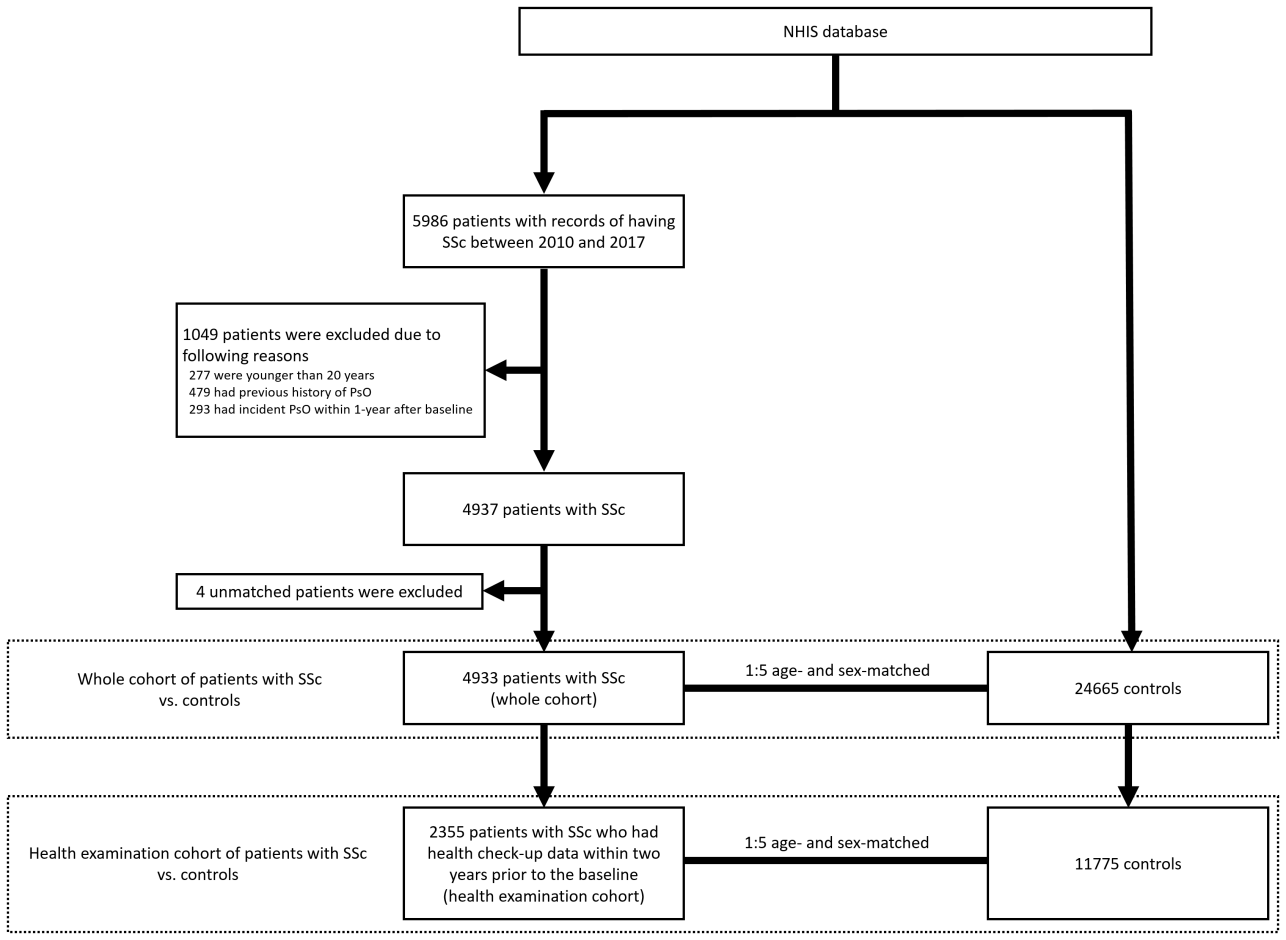
Selection of the study cohort and controls from the NHIS database. NHIS, National Health Insurance Service; SSc, systemic sclerosis; PsO, psoriasis.

From the NHIS database, we selected patients with records of having SSc between January 2010 and December 2017. SSc was defined as the RID code V138 (13). A total of 5,986 patients with SSc were identified. The exclusion criteria were as follows (1): age <20 years (n = 277) (2); previous history of PsO (n = 479) (3); incident PsO that occurred within 1 year from baseline (n = 293). After removing patients who met the exclusion criteria, the remaining 4,937 patients with SSc were used to select age- and sex-matched controls from the NHIS database at a ratio of 1:5. As a result, 4,933 patients with SSc (designated as the whole cohort) and 24,665 age- and sex-matched controls were included in the analysis (**Figure 1**).

All individuals were followed up for the occurrence of incident PsO until December 2019. We additionally analysed a subgroup of individuals who underwent national health check-ups within 2 years before the baseline (number of patients with SSc = 2,355 [designated as the health examination cohort]; and number of age- and sex-matched controls = 11,775). The health check-up data included anthropometric data, and laboratory data, such as serum levels of fasting glucose, cholesterol, and creatinine. Smoking status, alcohol consumption, and physical activity, which were based on standardised self-reporting questionnaires, were also included. These data were additionally considered in the analysis of this subgroup of individuals.

### Definition of PsO and covariates

PsO was defined as the ICD-10 code L40 (14). In the analysis of the whole cohort vs. controls, covariates were defined as follows: hypertension was defined as ICD-10 codes I10−I13 and I15 with prescriptions for antihypertensive agents; type 2 diabetes as ICD-10 codes E11–14 with prescriptions for anti-diabetic medications; dyslipidaemia as the ICD-10 code E78 with prescriptions for lipid-lowering agents; myocardial infarction (MI) as ICD-10 codes I21 or I22 during hospitalisation or these codes being recorded at least twice; stroke as ICD-10 codes I63 or I64 during hospitalisation with claims for brain magnetic resonance imaging or computed tomography; congestive heart failure (CHF) as the ICD-10 code I50; chronic obstructive pulmonary disease (COPD) as ICD-10 codes J41–J44; end-stage renal disease (ESRD) as a combination of ICD-10 codes (N18-19, Z49, Z94.0, and Z99.2) and an RID code assigned to patients with chronic kidney disease (CKD) that required haemodialysis (V001), peritoneal dialysis (V003), or a kidney transplantation (V005); and cancer as ICD-10 codes C00–C96 (7, 15–17).

In the separate analysis of the health examination cohort vs. controls, hypertension was defined as ICD-10 codes I10−I13 and I15 with prescriptions for antihypertensive agents or systolic or diastolic blood pressure ≥140 mmHg or ≥90 mmHg, respectively; type 2 diabetes as ICD-10 codes E11–14 and at least one annual claim of a prescription of anti-diabetic medications or fasting plasma glucose ≥126 mg/dL; dyslipidaemia as the ICD-10 code E78 with prescriptions for lipid-lowering agents or serum total cholesterol ≥240 mg/dL; and CKD as an estimated glomerular filtration rate (calculated using the Modification of Diet in Renal Disease equation) of <60 mL/min/1.73m^2^ (18). The following covariates were also considered in this analysis: smoking status (current vs. non-current smoker); alcohol consumption (alcohol intake per day >0 g: yes vs. no); physical activity (moderate exercise ≥5 days or vigorous exercise ≥3 days per week: yes vs. no); and obesity (BMI ≥25 kg/m^2^: yes vs. no).

### Statistical analysis

Continuous variables were expressed as mean ± standard deviation, and categorical variables were expressed as numbers (%). Continuous variables were compared using an independent Student’s *t*-test, and categorical variables were compared using the χ^2^ test. The incidence rate of PsO was expressed as the number of events per 1,000 person-years. The cumulative incidences of PsO in patients with SSc and controls were visualised using the Kaplan–Meier curve analysis and compared using the log-rank test. The hazard ratios (HRs) and 95% confidence intervals (CIs) were estimated using Cox proportional hazard models. The Cox proportional hazard models were first conducted comparing the whole cohort vs. controls, and subsequently comparing the health examination cohort vs. controls. In the whole cohort, patients with SSc were analysed as a whole and separately as prevalent (diagnosed with SSc before 2010) and incident cases (diagnosed with SSc between 2010 and 2017). Model 1 was a univariable analysis. Model 2 was adjusted for age, sex, income, hypertension, type 2 diabetes, and dyslipidaemia. Model 3 was additionally adjusted for MI, stroke, CHF, COPD, ESRD, and cancer. In the comparison of the health examination cohort vs. controls, model 1 was a univariable analysis. Model 2 was adjusted for age, sex, income, smoking status, alcohol consumption, physical activity, and obesity. Model 3 was additionally adjusted for hypertension, type 2 diabetes, dyslipidaemia, and CKD. Model 4 was further adjusted for MI, stroke, CHF, COPD, and cancer. All *p*-values were two-sided, and a p-value <0.05 was considered statistically significant. Statistical analyses were performed using SAS version 9.4 (SAS Institute, Cary, NC, USA).

## Results

### Baseline characteristics

The baseline characteristics of the 4,933 patients with SSc and a comparison with those of the age- and sex-matched controls (n = 24,665) are reported in **Table 1**. Of the 4,933 patients with SSc, 3,190 (64.7%) patients were newly diagnosed with SSc (incident cases), and the remaining 1,743 (35.3%) patients were diagnosed with SSc before 2010 (prevalent cases). The mean age of the patients with SSc was 54.1 ± 12.5 years, and most (85.9%) were females. Patients with SSc more commonly had underlying comorbidities, including hypertension (54.0% vs. 25.2%, p <0.001), dyslipidaemia (19.0% vs. 16.6%, p <0.001), MI (1.6% vs. 0.5%, p <0.001), CHF (5.1% vs. 1.4%, p <0.001), COPD (21.5% vs. 6.8%, p <0.001), ESRD (0.8% vs. 0.2%, p <0.001), and cancer (3.9% vs. 2.7%, p <0.001), than controls. In patients with SSc, although the follow-up duration was shorter (5.4 ± 2.8 years vs. 5.9 ± 2.7 years, p <0.001), the incidence of PsO was significantly higher (5.5% vs. 1.9%, p <0.001) than in controls.

**Table 1:** Baseline characteristics of the patients with systemic sclerosis and controls.

	Total population (N = 29,598)	Patients with SSc (N = 4,933)	Controls (N = 24,665)	P-value
Age, years	54.1 ± 12.5	54.1 ± 12.5	54.1 ± 12.5	>0.999
<65 years, n (%)	23,400 (79.1)	3,900 (79.1)	19,500 (79.1)	>0.999
≥65 years, n (%)	6,198 (20.9)	1,033 (20.9)	5,165 (20.9)	
Male sex, n (%)	4,188 (14.1)	698 (14.1)	3,490 (14.1)	>0.999
Female sex, n (%)	25,410 (85.9)	4,235 (85.9)	21,175 (85.9)	
Low income^a^, n (%)	7,374 (24.9)	1,210 (24.5)	6,164 (25.0)	0.493
Hypertension, n (%)	8,869 (30.0)	2,665 (54.0)	6,204 (25.2)	<0.001
Type 2 diabetes, n (%)	2,501 (8.4)	384 (7.8)	2,117 (8.6)	0.066
Dyslipidaemia, n (%)	5,024 (17.0)	935 (19.0)	4,089 (16.6)	<0.001
MI, n (%)	209 (0.7)	77 (1.6)	132 (0.5)	<0.001
Stroke, n (%)	178 (0.6)	34 (0.7)	144 (0.6)	0.382
CHF, n (%)	602 (2.0)	254 (5.1)	348 (1.4)	<0.001
COPD, n (%)	2,743 (9.3)	1,063 (21.5)	1,680 (6.8)	<0.001
ESRD, n (%)	84 (0.3)	37 (0.8)	47 (0.2)	<0.001
Cancer, n (%)	854 (2.9)	194 (3.9)	660 (2.7)	<0.001
Incident PsO, n (%)	735 (2.5)	272 (5.5)	463 (1.9)	<0.001
Follow-up duration, years	5.8 ± 2.7	5.4 ± 2.8	5.9 ± 2.7	<0.001

Continuous variables are expressed in mean ± SD.

a Lowest 25th percentile.

SSc, systemic sclerosis; MI, myocardial infarction; CHF, congestive heart failure; COPD, chronic obstructive pulmonary disease; ESRD, end-stage renal disease; PsO, psoriasis.

### Incidence rates and risk of incident PsO in patients with SSc (whole cohort) and controls

**Figure 2:**
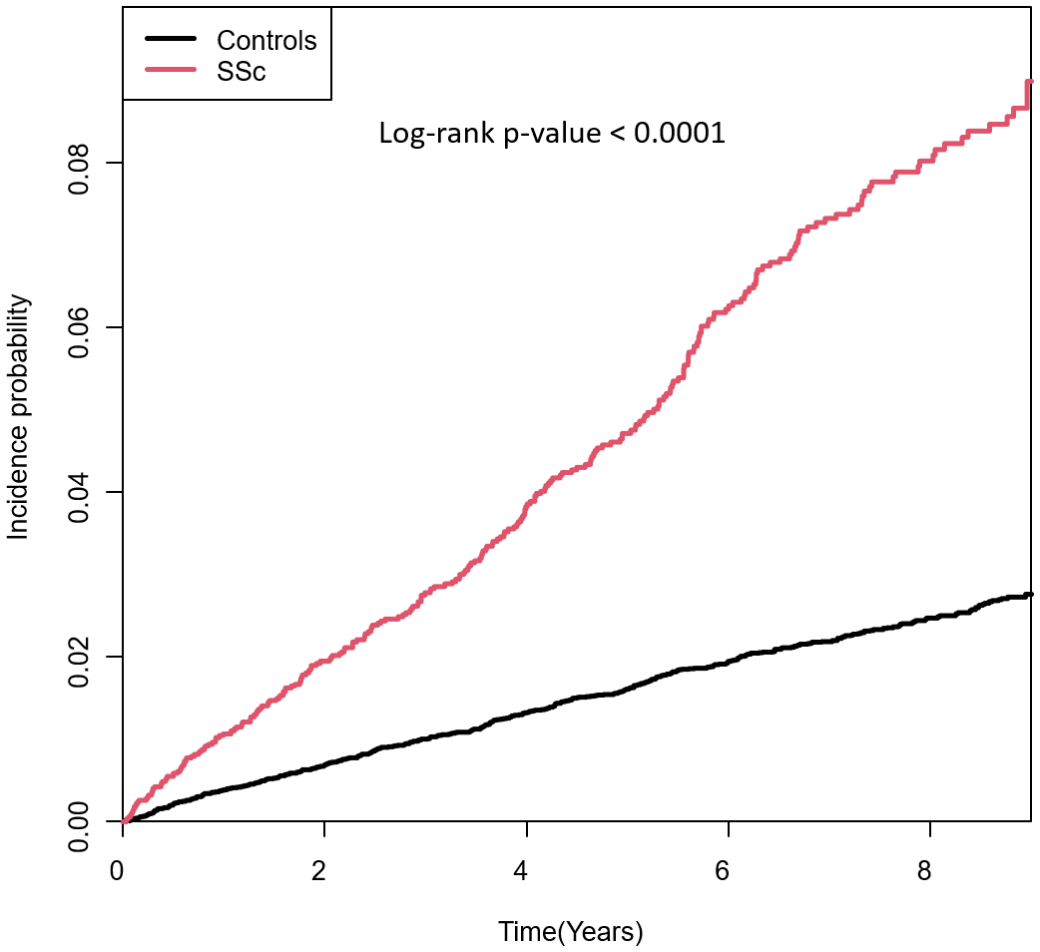
Cumulative incidences of PsO in patients with SSc and controls. SSc, systemic sclerosis; PsO, psoriasis.

The incidence rates of PsO in patients with SSc and controls were 10.26 and 3.20 per 1000 person-years, respectively. The cumulative incidences of PsO in patients with SSc and controls are visualised in **Figure 2**. Patients with SSc had a significantly higher cumulative incidence of PsO than controls (p <0.001).

In the univariable model, patients with SSc had a significantly higher risk of incident PsO (unadjusted HR: 3.198 [95% CI: 2.753, 3.715]). In the multivariable models adjusted for age, sex, income, and comorbidities, the higher risk of incident PsO in patients with SSc remained statistically significant (model 2, adjusted HR: 3.225 [95% CI: 2.753, 3.778]; model 3, adjusted HR: 3.055 [95% CI: 2.597, 3.594]) (**Table 2**). When patients with SSc were separately analysed as prevalent and incident cases, the significantly higher risk of incident PsO was observed in both the patients with prevalent SSc (model 3, adjusted HR: 3.455 [95% CI: 2.705, 4.414]) and incident SSc (model 3, adjusted HR: 2.748 [95% CI: 2.210, 3.417]) than in controls.

**Table 2:** Comparison of risk of incident psoriasis between patients with systemic sclerosis and controls.

	N	Incident PsO	Duration, pyrs	Incidence rate/ 1,000 pyrs	Model 1 HR (95% CI)	Model 2 HR (95% CI)	Model 3 HR (95% CI)
Comparison of all patients with SSc vs. controls							
Controls	24,665	463	144,671.7	3.20	1 (Ref.)	1 (Ref.)	1 (Ref.)
Patients with SSc	4,933	272	26,507.0	10.26	3.198 (2.753, 3.715)	3.225 (2.753, 3.778)	3.055 (2.597, 3.594)
Comparison of patients with prevalent cases of SSc^a^ vs. controls							
Controls	8,715	198	70,593.0	2.80	1 (Ref.)	1 (Ref.)	1 (Ref.)
Patients with SSc	1,743	131	12,656.6	10.35	3.670 (2.943, 4.577)	3.694 (2.915, 4.680)	3.455 (2.705, 4.414)
Comparison of patients with incident cases of SSc^b^ vs. controls							
Controls	15,950	265	74,078.8	3.58	1 (Ref.)	1 (Ref.)	1 (Ref.)
Patients with SSc	3,190	141	13,850.4	10.18	2.848 (2.322, 3.494)	2.866 (2.315, 3.548)	2.748 (2.210, 3.417)

Model 1: Non-adjusted.

Model 2: Adjusted for age, sex, income, hypertension, type 2 diabetes, and dyslipidaemia.

Model 3: Adjusted for age, sex, income, hypertension, type 2 diabetes, dyslipidaemia, MI, stroke, CHF, COPD, ESRD, and cancer.

a Patients diagnosed with SSc before 2010.

b Patients diagnosed with SSc after 2010.

PsO, psoriasis; pyrs, person-years; HR, hazard ratio; CI, confidence interval; SSc, systemic sclerosis; MI, myocardial infarction; CHF, congestive heart failure; COPD, chronic obstructive pulmonary disease; ESRD, end-stage renal disease.

### Incidence rates and risk of incident PsO in patients with SSc (health examination cohort) and controls

The characteristics of the 2,355 patients with SSc included in the health examination cohort and a comparison with those of the age- and sex-matched controls are shown in **Table 3**. Patients with SSc had a lower proportion of alcohol drinkers (23.2% vs. 26.6%, p = 0.001), less commonly performed regular physical activity (17.1% vs. 19.0%, p = 0.027), and less commonly had obesity (25.1% vs. 32.3%, p <0.001), type 2 diabetes (9.4% vs. 12.0%, p <0.001), and dyslipidaemia (27.9% vs. 31.1%, p = 0.002), compared with controls. In contrast, patients with SSc more commonly had hypertension (58.2% vs. 34.1%, p <0.001), MI (1.3% vs. 0.5%, p <0.001), CHF (4.8% vs. 1.4%, p <0.001), COPD (20.7% vs. 7.2%, p <0.001), and cancer (3.8% vs. 2.7%, p = 0.005) than controls. During a shorter follow-up duration than controls (4.8 ± 2.6 years vs. 5.2 ± 2.6 years, p <0.001), patients with SSc had a significantly higher incidence of PsO (4.7% vs. 1.8%, p <0.001).

**Table 3:** Baseline characteristics of the patients with systemic sclerosis and controls who had health check-up data.

	Total population (N = 14,130)	Patients with SSc (N = 2,355)	Controls (N = 11,775)	P-value
Age, years	55.8 ± 10.8	55.8 ± 10.8	55.8 ± 10.8	>0.999
<65 years, n (%)	11,022 (78.0)	1,837 (78.0)	9,185 (78.0)	>0.999
≥65 years, n (%)	3,108 (22.0)	518 (22.0)	2,590 (22.0)	
Male sex, n (%)	2,004 (14.2)	334 (14.2)	1,670 (14.2)	>0.999
Female sex, n (%)	12,126 (85.8)	2,021 (85.8)	10,105 (85.8)	
Low income^a^, n (%)	3,397 (24.0)	566 (24.0)	2,831 (24.0)	0.993
Current smoker, n (%)	1,084 (7.7)	170 (7.2)	914 (7.8)	0.366
Alcohol drinker, n (%)	3,681 (26.1)	547 (23.2)	3,134 (26.6)	<0.001
Regular physical activity, n (%)	2,642 (18.7)	402 (17.1)	2,240 (19.0)	0.027
Obesity, n (%)	4,397 (31.1)	592 (25.1)	3,805 (32.3)	<0.001
Hypertension, n (%)	5,387 (38.1)	1,371 (58.2)	4,016 (34.1)	<0.001
Type 2 diabetes, n (%)	1,636 (11.6)	222 (9.4)	1,414 (12.0)	<0.001
Dyslipidaemia, n (%)	4,320 (30.6)	656 (27.9)	3,664 (31.1)	0.002
CKD, n (%)	846 (6.0)	157 (6.7)	689 (5.9)	0.128
MI, n (%)	90 (0.6)	31 (1.3)	59 (0.5)	<0.001
Stroke, n (%)	78 (0.6)	15 (0.6)	63 (0.5)	0.542
CHF, n (%)	277 (2.0)	112 (4.8)	165 (1.4)	<0.001
COPD, n (%)	1,334 (9.4)	487 (20.7)	847 (7.2)	<0.001
ESRD, n (%)	16 (0.1)	3 (0.1)	13 (0.1)	0.823
Cancer, n (%)	408 (2.9)	89 (3.8)	319 (2.7)	0.005
BMI, kg/m^2^	23.7 ± 3.3	23.0 ± 3.2	23.8 ± 3.3	<0.001
Systolic BP, mmHg	121.3 ± 15.4	117.4 ± 15.2	122.0 ± 15.4	<0.001
Diastolic BP, mmHg	75.0 ± 10.0	72.9 ± 9.9	75.4 ± 10.0	<0.001
Fasting glucose, mg/dL	98.2 ± 23.3	95.0 ± 21.0	98.9 ± 23.7	<0.001
Total cholesterol, mg/dL	197.9 ± 38.1	188.2 ± 38.9	199.8 ± 37.7	<0.001
GFR, mL/min/1.73m^2^	89.2 ± 36.5	90.0 ± 33.9	89.0 ± 37.0	0.224
Incident PsO, n (%)	321 (2.3)	111 (4.7)	210 (1.8)	<0.001
Follow-up duration, years	5.1 ± 2.6	4.8 ± 2.6	5.2 ± 2.6	<0.001

Continuous variables are expressed in mean ± SD.

a Lowest 25th percentile.

SSc, systemic sclerosis; CKD, chronic kidney disease; MI, myocardial infarction; CHF, congestive heart failure; COPD, chronic obstructive pulmonary disease; ESRD, end-stage renal disease; BMI, body mass index; BP, blood pressure; GFR, glomerular filtration rate; PsO, psoriasis

The incidence rates of PsO in patients with SSc and controls were 9.74 and 3.44 per 1000 person-years, respectively. In the univariable model, patients with SSc had a significantly higher risk of incident PsO (unadjusted HR: 2.821 [95% CI: 2.241, 3.551]). In model 2, which was adjusted for age, sex, income, smoking, alcohol consumption, physical activity, and obesity, patients with SSc showed a significantly higher risk of incident PsO (adjusted HR: 2.872 [95% CI: 2.279, 3.618]). This association remained statistically significant in the models which additionally adjusted for comorbidities (model 3, adjusted HR: 2.941 [95% CI: 2.315, 3.737]; model 4, adjusted HR: 2.820 [95% CI: 2.207, 3.603]; **Table 4**).

**Table 4:** Comparison of risk of incident psoriasis between patients with systemic sclerosis and controls who had health check-up data.

	N	Incident PsO	Duration, pyrs	Incidence rate/ 1,000 pyrs	Model 1 HR (95% CI)	Model 2 HR (95% CI)	Model 3 HR (95% CI)	Model 4 HR (95% CI)
Controls	11,775	210	60,965.5	3.44	1 (Ref.)	1 (Ref.)	1 (Ref.)	1 (Ref.)
Patients with SSc	2,355	111	11,391.9	9.74	2.821 (2.241, 3.551)	2.872 (2.279, 3.618)	2.941 (2.315, 3.737)	2.820 (2.207, 3.603)

Model 1: Non-adjusted

Model 2: Adjusted for age, sex, income, smoking status, alcohol consumption, physical activity, and obesity

Model 3: Adjusted for age, sex, income, smoking status, alcohol consumption, physical activity, obesity, hypertension, type 2 diabetes, dyslipidaemia, and CKD

Model 4: Adjusted for age, sex, income, smoking status, alcohol consumption, physical activity, obesity, hypertension, type 2 diabetes, dyslipidaemia, CKD, MI, stroke, CHF, COPD, and cancer

PsO, psoriasis; pyrs, person-years; HR, hazard ratio; CI, confidence interval; SSc, systemic sclerosis; CKD, chronic kidney disease; MI, myocardial infarction; CHF, congestive heart failure; COPD, chronic obstructive pulmonary disease.

## Discussion

In this study, we assessed whether SSc contributes as a risk factor of incident PsO and found that patients with SSc are at a higher risk of developing incident PsO than age- and sex-matched controls without SSc. The analyses were adjusted for known risk factors of PsO, suggesting SSc as an independent risk factor of incident PsO. To the best of our knowledge, this is the first study to evaluate SSc as an independent risk factor of incident PsO.

The analyses were performed in two parts. First, the whole cohort of patients with SSc and their matched controls were analysed. Second, patients with SSc who underwent health check-ups within two years before the baseline (health examination cohort) and their matched controls were analysed. In the first part of the analysis, 4,933 patients with SSc were included, which is one of the largest studies on patients with SSc. Although this part of the analysis was strengthened by the large sample size, data on smoking and alcohol consumption, which are risk factors of PsO (10), were not available and, therefore, not adjusted. In the second part of the analysis, using the health check-up data, we were able to further adjust for additional risk factors of PsO, including smoking, alcohol consumption, and obesity (10). Moreover, we used operational definitions of hypertension, type 2 diabetes, and dyslipidaemia different from that used in the whole cohort. Although the sample size (number of patients with SSc = 2,355) was reduced compared with the whole cohort, the second part of the analysis had strength because it adjusted for additional known risk factors of PsO and used different definitions of some covariates, which tests the robustness of the findings observed in the first part of the analysis. Both analyses revealed similar results: adjusted HR was 3.055 (95% CI: 2.597, 3.594) in the first part of the analysis (model 3) and 2.820 (95% CI: 2.207, 3.604) in the second part (model 4), adding robustness to our findings.

In the first part of the analysis, in addition to analysing patients with SSc as a whole, we classified the patients with SSc into prevalent and incident cases and analysed them separately. These analyses were conducted to evaluate whether there is a period effect between patients diagnosed with SSc before 2010 (prevalent cases of SSc) and those diagnosed with SSc after 2010 (incident cases of SSc). The effect size was larger in patients with prevalent cases of SSc (model 3, adjusted HR: 3.455 [95% CI: 2.705, 4.414]) than in those with incident cases of SSc (model 3, adjusted HR: 2.748 [95% CI: 2.210, 3.417]). A possible explanation for the larger effect size in patients with prevalent cases of SSc could be the difference in exposure to angiotensin-converting enzyme inhibitor (ACEi), which is the drug of choice for treating scleroderma renal crisis (SRC) (19, 20). ACEi is widely accepted as a medication that provokes PsO (21, 22). A trend of decrease in the frequency of SRC over time has been reported in a meta-analysis (23). Although there seems to be a period effect between prevalent and incident cases of SSc, it is important to note that the higher risk of incident PsO was consistently observed in both the patients with prevalent cases of SSc and those with incident cases of SSc.

Nailfold capillary changes are one of the characteristic findings of SSc (1). Interestingly, a recent study has reported that abnormal nailfold capillary changes are also seen in patients with PsO, and are risk factors for progression to psoriatic arthritis (24). It is intriguing that nailfold capillary changes can be observed in both SSc and PsO. The similarities between SSc and PsO indicate that the two diseases are indeed closely related.

T helper 17 (Th17) cells and interleukin (IL)-17 are well-known to play a central role in the pathogenesis of PsO (25, 26). A large body of evidence suggests that Th17 response is also involved in the pathogenesis of SSc (27, 28). Importantly, a study has reported that B cells differentiate CD4+ T cells into Th17 T cells in SSc (29). Furthermore, studies have shown that serum levels of IL-17 and the mRNA expression levels of IL-17 in skin lesions are higher in patients with SSc than in healthy controls (30, 31). Another study reported that the levels of circulating Th17 cells are higher in patients with SSc than in controls and correlate with disease severity (32). Mechanistically, Th17 cell-derived IL-17 is involved in fibroblast growth and collagen overproduction (32). Collectively, Th17 cells play an important role in both SSc and PsO. The shared pathogenesis between SSc and PsO could be a possible explanation for why patients with SSc showed a higher risk of incident PsO than controls.

From a therapeutic perspective, given that Th17 cells play a crucial role in both SSc and PsO, targeting IL-23–Th17 pathway could be useful for both SSc and PsO. Indeed, guselkumab (an IL-23 inhibitor), secukinumab (an IL-17A inhibitor), ixekizumab (an IL-17A inhibitor), and brodalumab (an IL-17 receptor A inhibitor) are approved for the treatment of PsO (33). Moreover, treatments targeting IL-23–Th17 pathway are also gaining attention in SSc recently (34, 35). A single-arm, open-label, phase 1 trial has reported that brodalumab is efficacious in decreasing modified Rodnan skin score in patients with SSc (34). With regard to treatment targeting IL-23, a case series of three patients with SSc who also had PsO has shown a promising result (35). Guselkumab was not only effective in improving PsO, but also effective in improving immune abnormalities, fibrosis, and vasculopathy, which are the three components of SSc (35). These findings reflect the importance of IL-23–Th17 pathway as a potential therapeutic target in both diseases.

Another possible explanation for the higher risk of incident PsO in patients with SSc is that skin damage, such as digital tip ulcers caused by SSc (1), may have led to the exposure of autoantigens to the immune system and triggered an aberrant immune reaction. Indeed, aberrant activation of tissue-resident memory T cells in response to autoantigens is implicated in the pathogenesis of PsO (36).

It is possible that patients with SSc are more likely to be seeing a dermatologist than controls, which could lead to a higher detection of PsO in patients with SSc. In other words, the possibility of under-detection of PsO in controls due to not seeing a dermatologist should be considered. A population-based study assessing diagnostic delay of PsO has reported that the time from symptom onset to being seen by a dermatologist is approximately 3 years (male, 3.9 years; and female, 2.7 years) (37). Given that the mean follow-up duration was 5.9 ± 2.7 years in the control group of our study, it is unlikely that PsO would have been under-detected in the controls due to not seeing a dermatologist.

There are some limitations to our study. First, this was a retrospective observational study. Although we adjusted for multiple confounders, residual confounding cannot be fully excluded. Second, data regarding autoantibody profile, organs involved, and disease severity of SSc are not available in the NHIS database, and consequently, we could not assess whether there is a specific subset of patients with SSc who are particularly at a higher risk of incident PsO. Third, as only Koreans were included in our study, the results might not be generalised to population of other ethnicities. However, using the NHIS database, which covers the entire Korean population, we were able to analyse a large number of patients with SSc, which is a rare disease. This allowed us to assess the risk of incident PsO in this rare disease, that could not be evaluated in clinical trials, which usually include a relatively small number of patients.

In conclusion, this large population-based cohort study showed that patients with SSc have a 3-fold higher risk of developing incident PsO than controls. This association was significant after adjusting for known risk factors of PsO, suggesting that SSc could be a possible independent risk factor of incident PsO.

## Data availability statement

The original contributions presented in the study are included in the article/supplementary material. Further inquiries can be directed to the corresponding authors.

## Ethics statement

This study was approved by the Institutional Review Board (IRB) of Gangnam Severance Hospital (IRB No: 3-2022-0338). Owing to the retrospective nature of this study, the requirement for informed consent was waived and approved by the IRB of Gangnam Severance Hospital.

## Author contributions

OCK: Conceptualization, Data curation, Funding acquisition, Investigation, Methodology, Project administration, Resources, Software, Validation, Visualization, Writing – original draft. KH: Conceptualization, Data curation, Formal Analysis, Investigation, Methodology, Project administration, Resources, Software, Supervision, Validation, Visualization, Writing – review & editing. M-CP: Conceptualization, Data curation, Investigation, Methodology, Project administration, Resources, Software, Supervision, Validation, Visualization, Writing – review & editing.
